# Editorials

**Published:** 1897-11

**Authors:** 


### HOMŒOPATHIC PHYSICIAN,

A MONTHLY JOURNAL OF

HOMŒOPATHIC MATERIA MEDICA AND CLINICAL MEDICINE.

"If our school ever gives up the strict inductive method of Hahnemann, we are lost, and deserve only to be mentioned as a caricature in the history of medicine."—CONSTANTINE HERING.

Vol. XVII. NOVEMBER, 1897. No. 11.

### EDITORIALS.

THE NOTES ON DR. LIPPE'S LECTURES will be omitted this month because of the Editor's inability to properly prepare them. We hope to give them as usual next month.

---

VACCINATION.—The Editor hopes the profession continues to take an interest in the revelations concerning vaccination made by Dr. Leverson in this journal every month. We feel that it is a subject of the highest importance, and every word published should be carefully read and pondered. Only the most important and striking portions of the testimony are here reproduced, and if it seems to some of our readers as if too much space were devoted to it, we can assure them that the bulk of material from which it is drawn is simply enormous, and we have published only what is most remarkable for its glaring inconsistencies. The testimony of those who favor vaccination is nothing compared to what is to come, when the testimony of its opponents comes to be analyzed by the master mind of Dr. Leverson. The revelations *then* given will be astonishing, and must command the attention of even the most ardent advocates of the "rite."

